# Insecticidal activity and underlying molecular mechanisms of a phytochemical plumbagin against *Spodoptera frugiperda*

**DOI:** 10.3389/fphys.2024.1427385

**Published:** 2024-06-21

**Authors:** Xiaoyu Sun, Wenxuan Li, Shuang Yang, Xueqi Ni, Shengjie Han, Mengting Wang, Cong’ai Zhen, Xinzheng Huang

**Affiliations:** ^1^ Department of Entomology, MOA Key Lab of Pest Monitoring and Green Management, College of Plant Protection, China Agricultural University, Beijing, China; ^2^ College of Food Science and Nutritional Engineering, China Agricultural University, Beijing, China; ^3^ College of Horticulture and Plant Protection, Henan University of Science and Technology, Luoyang, China

**Keywords:** *Spodoptera frugiperda*, plumbagin, RNA-seq, detoxification enzyme, toxicological mechanism

## Abstract

**Introduction:**

Plumbagin is an important phytochemical and has been reported to exhibit potent larvicidal activity against several insect pests, However, the insecticidal mechanism of plumbagin against pests is still poorly understood. This study aimed to investigate the insecticidal activities of plumbagin and the underlying molecular mechanisms against a devastating agricultural pest, the fall armyworm *Spodoptera frugiperda*.

**Methods:**

The effects of plumbagin on *S. frugiperda* larval development and the activities of two detoxification enzymes were initially examined. Next, transcriptomic changes in *S. frugiperda* after plumbagin treatment were investigated. Furthermore, RNA-seq results were validated by qPCR.

**Results:**

Plumbagin exhibited a high larvicidal activity against the second and third instar larvae of *S. frugiperda* with 72 h LC_50_ of 0.573 and 2.676 mg/g, respectively. The activities of the two detoxification enzymes carboxylesterase and P450 were significantly increased after 1.5 mg/g plumbagin treatment. Furthermore, RNA-seq analysis provided a comprehensive overview of complex transcriptomic changes in *S. frugiperda* larvae in response to 1.5 mg/g plumbagin exposure, and revealed that plumbagin treatment led to aberrant expression of a large number of genes related to nutrient and energy metabolism, humoral immune response, insect cuticle protein, chitin-binding proteins, chitin synthesis and degradation, insect hormone, and xenobiotic detoxification. The qPCR results further validated the reproducibility and reliability of the transcriptomic data.

**Discussion:**

Our findings provide a valuable insight into understanding the insecticidal mechanism of the phytochemical plumbagin.

## 1 Introduction

The fall armyworm, *Spodoptera frugiperda* (J.E. Smith), a migratory, highly polyphagous, and widely distributed destructive lepidopteran pest species, is native to tropical and subtropical areas of the Americas (Kenis et al., 2023). Since 2016, this pest has invaded West Africa, and then rapidly spread to sub-Saharan Africa nations, Asia, and parts of Oceania such as southern Australia (Kenis et al., 2023; Tay et al., 2023). In China, *S*. *frugiperda* was first observed in January 2019 from maize field in Jiangcheng County, Yunnan Province (Guo et al., 2023; Sun et al., 2021), and then quickly spread to 27 provinces (autonomous regions and municipalities) across China, and thus being considered to be a major invasive crop pest posing a significant threat to crop production and agricultural food security (Wang et al., 2023). Generally, most farmers and agricultural practices in many invasive regions have relied primarily on the application of synthetic pesticides to manage *S*. *frugiperda*. Unfortunately, their improper application and excessive utilization have brought various negative effects such as nontarget toxicity, pesticide residue accumulation in agricultural products and the environment, and resistance of this pest to spinosad, diamides and *Bacillus thuringiensis* (Zhao et al., 2022; Tay et al., 2023). Over the past few years, significant progress has been made in the development of effective and sustainable management strategies to control this devastating pest in many recently invaded countries, including China. For example, effective and eco-friendly botanical pesticides have received increasing attention in sustainable agriculture, and are recognized as one of the most promising alternatives to synthetic insecticides for the development and practical implementation of integrated pest management programmes (Kenis et al., 2023).

Plumbagin, 5-hydroxy-2-methyl-1,4-naphthoquinone (C_11_H_8_O_3_), is an important secondary metabolite and bioactive compound in plants originally isolated from the medicinal plant *Plumbago zeylanica* and has been reported to exhibit potent larvicidal activity against several insect pests, including *S. litura* (Tokunaga et al., 2004; Rajendra Prasad et al., 2012), *Achaea Janata* (Rajendra Prasad et al., 2012), *Trichoplusia ni* (Akhtar et al., 2012a), *Musca domestica* (Pavela, 2013), *Helicoverpa armigera* (Hu et al., 2018), *Pieris rapae* (Hu et al., 2018), *Mythimna separate* (Shang et al., 2019), *Nilaparvata lugens* (Shang et al., 2019), *S. littoralis* (Davila-Lara et al., 2021; Rahman-Soad et al., 2021), *Aedes aegypti* (de Oliveira et al., 2022), and three aphid species (Akhtar et al., 2012b), as well as acaricidal activity against the herbivorous mite *Tetranychus urticae* (Akhtar et al., 2012b). The results of these studies have indicated that plumbagin has great potential to be developed as a potent botanical insecticide and an alternative to synthetic insecticides. However, little is known about the acute toxicity of plumbagin on *S. frugiperda* and the underlying molecular mechanisms of larvae in response to plumbagin exposure.

Recently, rapid and remarkable developments in sequencing technology and increasing numbers of complete insect genomes have opened up new avenues for exploring global transcriptome changes reflecting the physiological state of insect pests to plant metabolites exposures, unveiling previously unknown mechanisms and mining functional genes (Lin et al., 2023; Li et al., 2023). Huang et al. (2018) used RNA-Seq and qPCR to characterize the expression levels of detoxification genes, especially P450 genes in *Sitophilus zeamais* responding to terpinen-4-ol, the main constituent of *Melaleuca alternifolia* essential oil. Li et al. (2023) analyzed transcriptome profiling of linalool-exposed *Pagiophloeus tsushimanus* larvae using RNA-seq and single-molecule real-time sequencing. Gene ontology enrichment of DEGs revealed that overall upregulation of DEGs encoding cytochrome P450s and cuticular proteins was the primary response characteristic in larvae upon exposure to linalool. The effects of several plant metabolites such as carvacrol (Liu et al., 2023), toosendanin (Lin et al., 2023), camptothecin (Shu et al., 2021a; Shu et al., 2021b), azadirachtin (Shu et al., 2021c), and chlorogenic acid (Lin et al., 2023) on gene expression profiles and enzyme activities in *S*. *frugiperda* larvae have recently been studied using RNA-seq and enzyme activity assays in order to elucidate the response mechanism. These findings indicated that these metabolites influenced the normal physiological activities of *S*. *frugiperda* at multiple levels.

In this study, the effects of plumbagin on *S. frugiperda* larval development and on the activities of two detoxification enzymes were examined to assess its bioactivity against this devastating agricultural pest. Next, transcriptomic changes of *S. frugiperda* after plumbagin treatment were investigated to elucidate the underlying molecular mechanism of its insecticidal activity. Furthermore, RNA-seq results were confirmed by qPCR. Our results will provide a better understanding of the toxicological mechanism of plumbagin used as a botanical pesticide against pests.

## 2 Material and methods

### 2.1 Insect

Eggs of *S*. *frugiperda* used in this study were supplied by the Institute of Plant Protection, Chinese Academy of Agricultural Sciences, which were collected from maize fields in Jiangcheng, Yunnan Province, China. The larvae were reared on artificial diet in a growth room at 26°C ± 1°C, 70% ± 5% relative humidity, with a photoperiod of 14:10 (L:D) as described previously (Su et al., 2023).

### 2.2 Bioassays and enzyme activity assays

Plumbagin (CAS number: 481-42-5) was purchased from Sigma-Aldrich. Dissolved in acetone, plumbagin was incorporated into the artificial diet at various concentrations of 3, 1.5, 0.75, 0.375, 0.1875, 0.09375 mg/g. Artificial diet incorporating acetone was used as control. A single second or third instar *S. frugiperda* larvae was placed in petri dish (35 mm in diameter × 2 cm in height) and reared on 1 g artificial diet. They were allowed to feed for 7 days, and mortality was recorded. Three biological replicates, each comprising a total of 20 larvae, were analyzed. The larval weight of surviving *S. frugiperda* was also recorded daily for 4 days.

Ten third instar larvae fed on diet containing 1.5 mg/g plumbagin were homogenized in 1 mL phosphate buffer solution. The homogenate was centrifuged for 20 min (13,000 rpm, 4°C), and the supernatants were collected for further enzyme activity assays. The enzyme activities of two detoxification enzymes carboxylesterase (CarE) and P450 were determined using the Carboxylesterase Test Kit (A133-1-1) and the Cytochrome P450 Assay Kit (H303-1-2) from Nanjing Jiancheng Bioengineering Institute (Jiangsu, China) following the manufacturer’s protocol.

### 2.3 RNA isolation and illumina sequencing

Third-instar *S*. *frugiperda* larvae were provided with an artificial diet incorporating 1.5 mg/g plumbagin as described above for bioassays. The larvae fed with an artificial diet containing acetone were used as controls. After 3 days, the survival larvae were collected and then subjected to RNA extraction and RNA sequencing analysis. Total RNA extraction was performed using TRIzol reagent (Invitrogen) following the manufacturer’s instructions. At least four biological replicates were analyzed for each treatment.

RNA purity was evaluated using the NanoPhotometer^®^ spectrophotometer (IMPLEN, CA, United States). A total of 3 μg RNA per sample was utilized for cDNA library preparation with the NEBNext Ultra RNA Library Prep Kit according to the manufacturer’s instructions. Samples were sequenced on an Illumina NovaSeq6000 platform (Biomics Biotech Co., Ltd., Beijing, China). Clean reads were aligned to the chromosome-level assembled genome of *S. frugiperda* available at http://v2.insect-genome.com/Organism/715 using TopHat v2.0.12 software. Gene Ontology (GO) and KEGG pathway enrichment analyses for differentially expressed genes (DEGs) were conducted using the GOseq R package and KOBAS software, respectively. Genes with a log_2_|fold change| > 1 and FDR < 0.05 were considered as DEGs.

### 2.4 qPCR validation

The qPCR validation was carried out based on SuperReal PreMix Plus (SYBR Green) reagent kit (TianGen, Beijing, China), according to the manufacturer’s instructions (Huang et al., 2018) and using the same samples used for RNA-seq. The qPCR programme was 95°C for 10 min, followed by 40 cycles of 95°C for 15 s and 60°C for 30 s. The gene β-actin was used as a reference gene as described previously (Su et al., 2023). The primers for qPCR were listed in Supplementary Table S1.

### 2.5 Statistical analysis

POLO Plus software was used to calculate the median lethal concentration (LC_50_), with corresponding 95% confidence interval (CI) and chi-square (*χ* ^2^) of the probit regression equation. For data on larval weight and enzyme activity assay, statistical analysis was performed using one-way analysis of variance (ANOVA) with LSD/Duncan pairwise comparison testing.

## 3 Results

### 3.1 Bioactivity of plumbagin against *Spodoptera frugiperda* larvae

**FIGURE 1:**
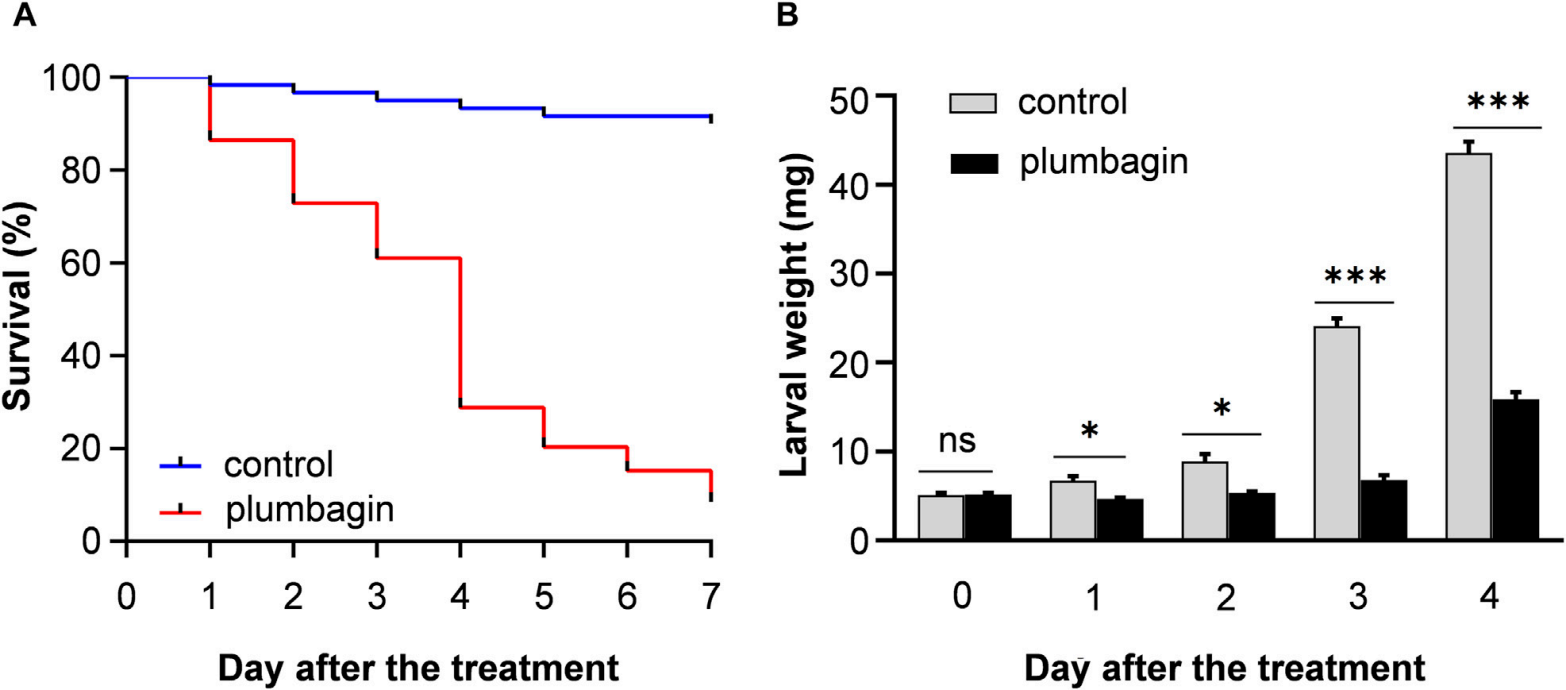
The survival rate **(A)** and larval weight **(B)** of *S. frugiperda* after 1.5 mg/g plumbagin treatment.

The toxicity of plumbagin to *S. frugiperda* was determined using second or third instar larvae. The results showed that plumbagin exhibited significant insecticidal activity against *S. frugiperda* larvae, and the LC_50_ values for second instar and third instar were 0.573 and 2.676 mg/g, respectively (Table 1). The mortality rate of 1.5 mg/g plumbagin treatment after 7 days was 91.53% (Figure 1A). The survival larvae during 1.5 mg/g plumbagin treatment displayed lower weight compared to controls, with weight reduction from 43.57 to 15.90 mg after 4 days (Figure 1B).

**TABLE 1:** Toxicity of plumbagin against *S. frugiperda* larvae after 72 h of treatment.

Larval stage	Slope ± SE^a^	LC_50_ (95% CI)^b^ mg/g	*χ* ^2^ (df)	*p*-value
2nd	2.690 ± 0.346	0.573 (0.471–0.696)	13.500 (13)	0.410
3rd	1.362 ± 0.243	2.676 (1.800–5.528)	13.781 (13)	0.389

^a^ SE, standard error.

^b^ 95% CI, 95% confidence intervals.

### 3.2 Enzyme activities of two detoxification enzymes in *S. frugiperda* larvae after plumbagin exposure

**FIGURE 2:**
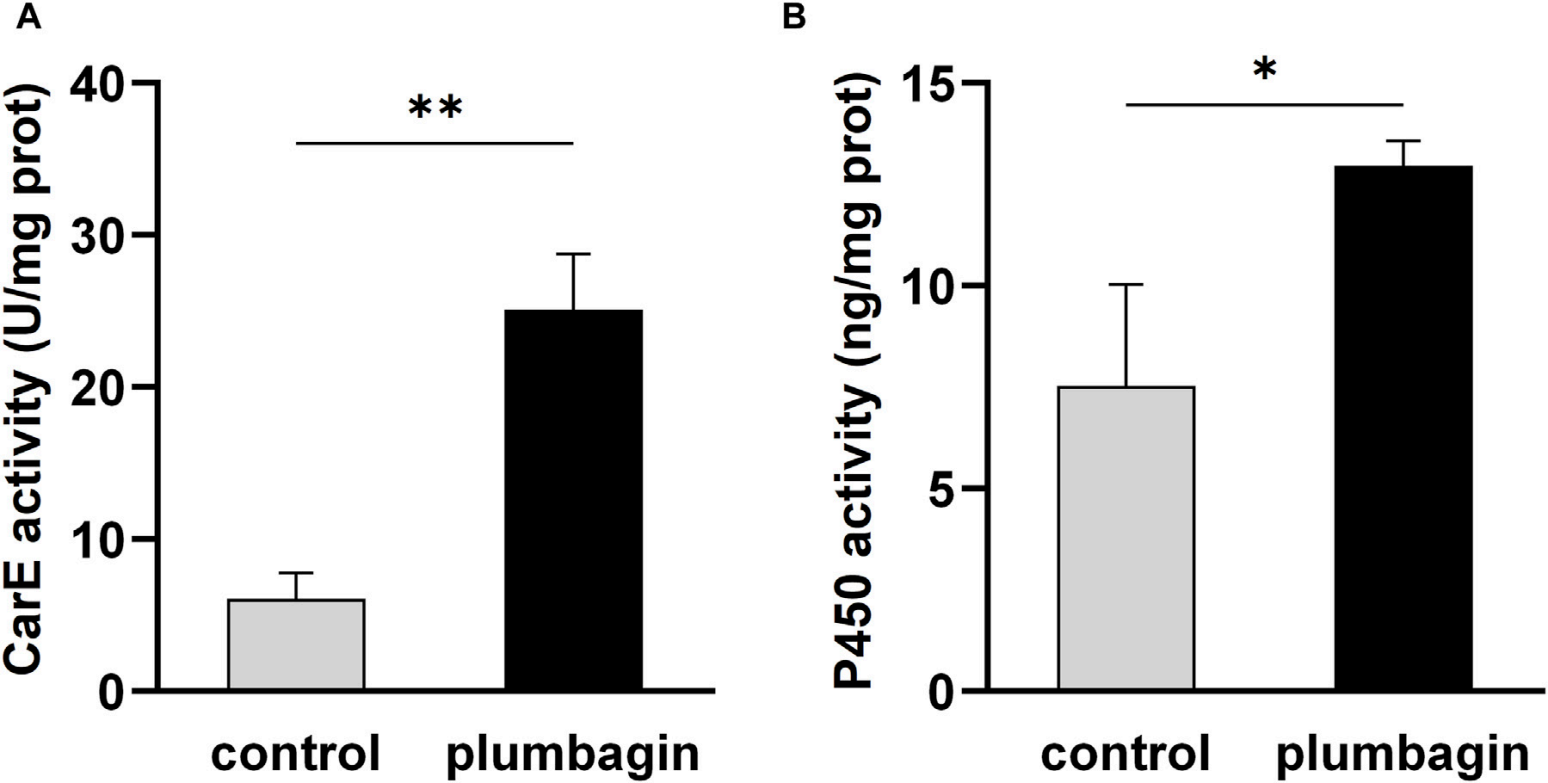
The activities of two detoxification enzymes in third-instar *S. frugiperda* larvae after 1.5 mg/g plumbagin treatment. **(A)** Carboxylesterase (CarE) activity. **(B)** P450 activity.

Furthermore, enzyme activity assays revealed that 1.5 mg/g plumbagin treatment significantly improved the activities of the two detoxification enzymes CarE and P450 compared with the control, with a 4.13- and 1.72-fold increase, respectively (Figure 2).

### 3.3 Global transcriptome changes in *S. frugiperda* larvae after plumbagin exposure

**FIGURE 3:**
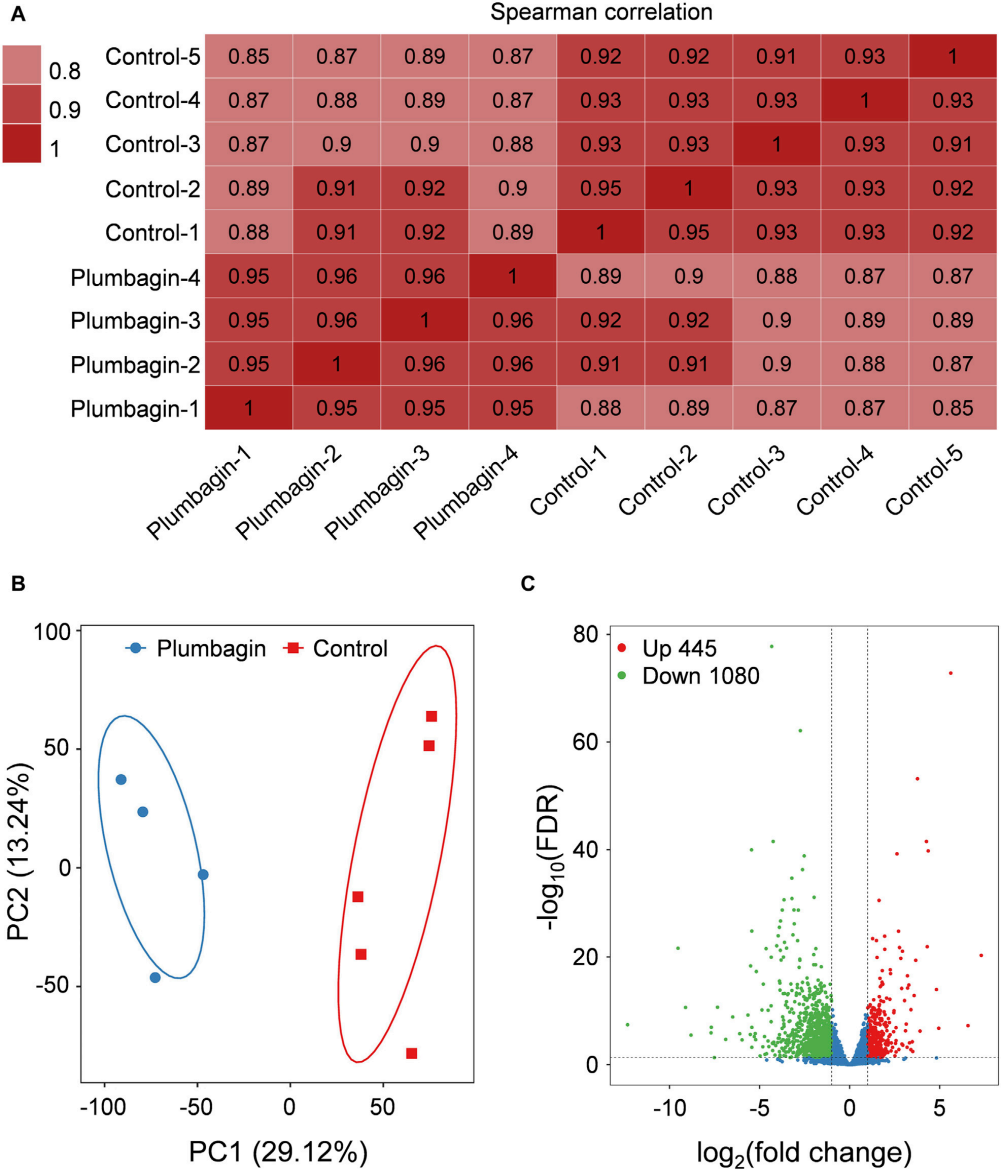
Transcriptome profile of RNA-Seq data. **(A)** Correlation matrix of the samples utilizing Spearman’s correlation coefficients. **(B)** Principal component analysis of the samples. **(C)** Volcano plot of RNA-Seq data.

Transcriptome sequencing of *S. frugiperda* larvae upon plumbagin exposure was conducted to explore the molecular mechanisms underlying the larvicidal activity of plumbagin. Nine independent cDNA libraries were generated, comprising four for the plumbagin treatment group and five for the control group. The raw reads number ranged from 41,489,334 to 45,978,878, while high quality clean reads varied between 40,213,076 and 45,148,130. The proportion of mapped clean reads in each sample ranged from 77.56% to 80.91% (Supplementary Table S2). Correlation within each group of experimental and control samples were generally high (Figure 3A). Principal component analysis (PCA) showed that the plumbagin-exposed samples and controls formed distinct clusters, indicating plumbagin exposure led to aberrant expression of genes in *S. frugiperda* larvae (Figure 3B).

### 3.4 Functional category of plumbagin-responsive DEGs in *S. frugiperda* larvae

After plumbagin exposure, a total of 1,614 genes with a log_2_|fold change| > 1 and FDR < 0.05 were identified as differentially expressed genes (DEGs) in *S. frugiperda* larvae. Among these DEGs, 445 were upregulated and 1,080 were downregulated (Figure 3C).

**FIGURE 4:**
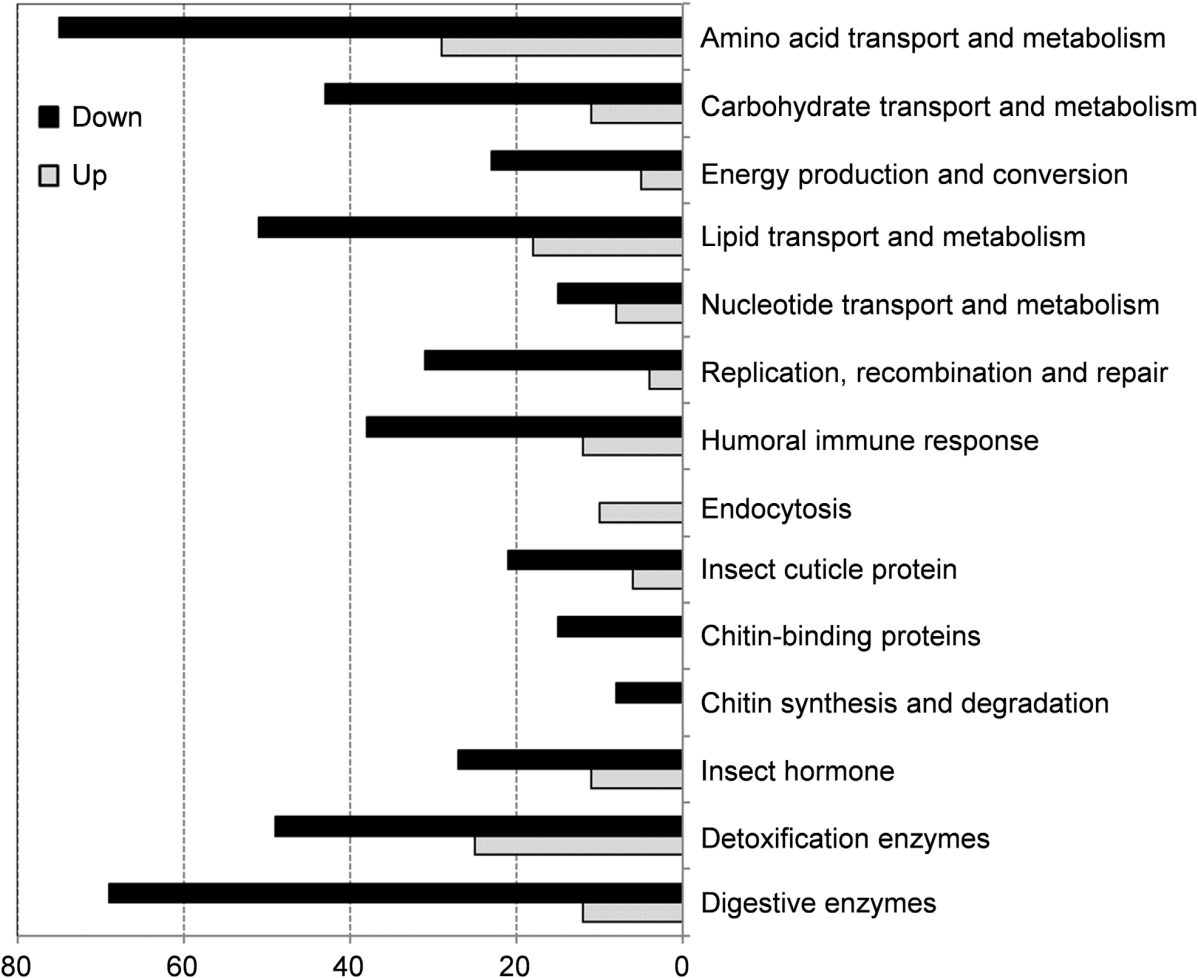
Enrichment analyses of differentially expressed genes (DEGs) in *S. frugiperda* larvae after 1.5 mg/g plumbagin treatment.

**FIGURE 5:**
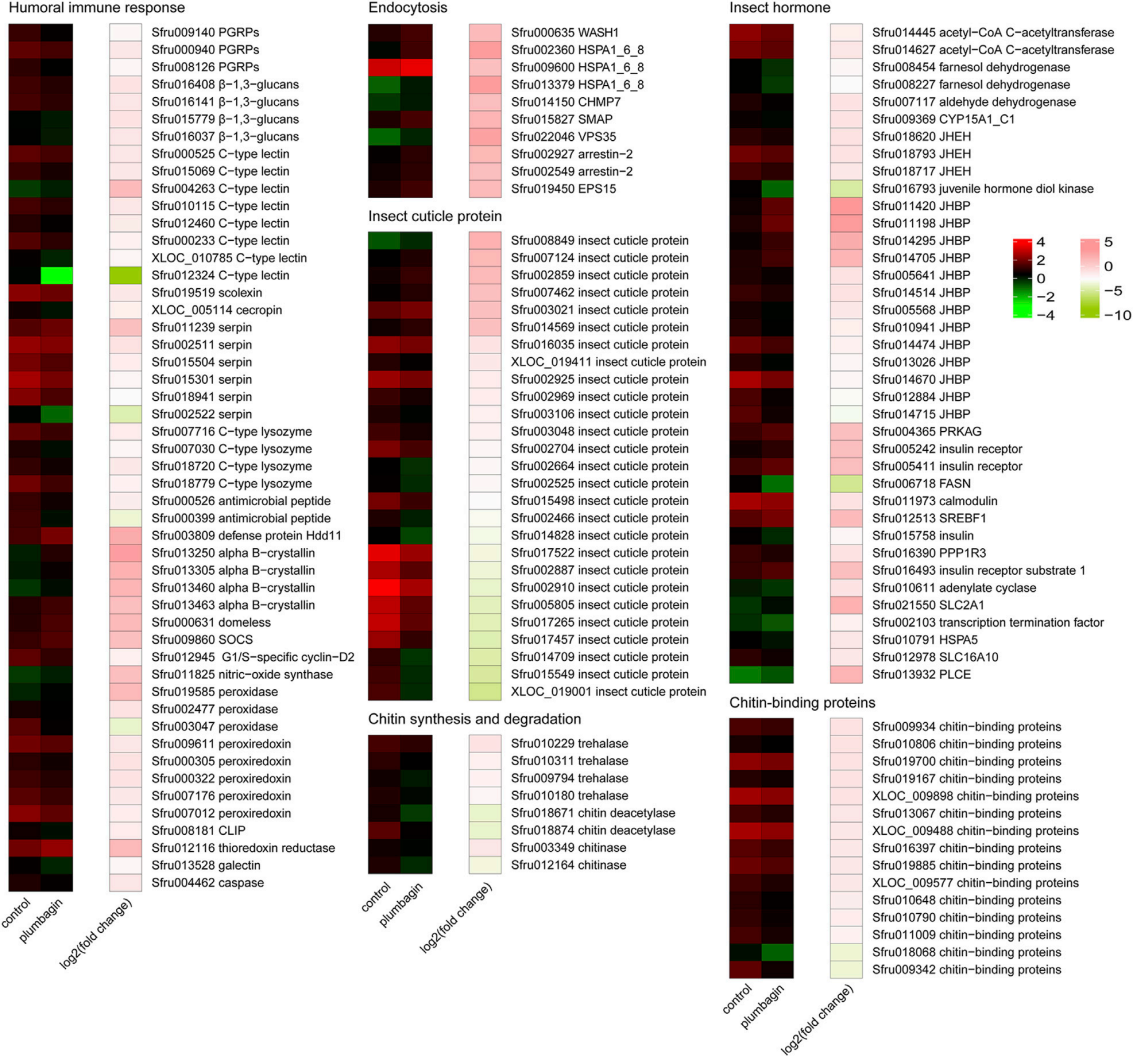
Heatmap analysis of differentially expressed genes (DEGs) in *S. frugiperda* larvae after plumbagin treatment. CLIP, clip domain serine protease; SOCS, suppressor of cytokine signaling 2; PGRPs, peptidoglycan recognition proteins; CHMP7, charged multivesicular body protein 7; HSPA1_6_8, heat shock 70 kDa protein 1/2/6/8; SMAP, stromal membrane-associated protein; VPS35, vacuolar protein sorting-associated protein 35; EPS15, epidermal growth factor receptor substrate 15; JHEH, juvenile hormone epoxide hydrolase; JHBP, haemolymph juvenile hormone binding protein; PRKAG, 5′-AMP-activated protein kinase, regulatory gamma subunit; FASN, fatty acid synthase, animal type; SREBF1, sterol regulatory element-binding transcription factor 1; PPP1R3, protein phosphatase 1 regulatory subunit 3A/B/C/D/E; SLC2A1, solute carrier family 2 (facilitated glucose transporter), member 1; HSPA5, heat shock 70 kDa protein 5; SLC16A10, solute carrier family 16 (monocarboxylic acid transporters), member 10; PLCE, phosphatidylinositol phospholipase C, epsilon; CYP15A1_C1, methyl farnesoate epoxidase/farnesoate epoxidase; WASH1, WAS protein family homolog 1.

Functional enrichment analysis including GO annotation enrichment, KOG functional enrichment and KEGG pathway analysis were conducted to explore the functional categories of these DEGs (Figures 4, 5), which showed that the plumbagin treatment exhibited profound strong adverse effects on many essential genes involved in amino acid transport and metabolism (75 out of 104 DEGs), carbohydrate transport and metabolism (43 out of 54 DEGs), energy production and conversion (23 out of 28 DEGs), lipid transport and metabolism (51 out of 69 DEGs), nucleotide transport and metabolism (15 out of 23 DEGs), and replication, recombination and repair (31 out of 35 DEGs). Also, most DEGs related to humoral immune response (38 out of 50 DEGs), insect cuticle protein (21 out of 27 DEGs), insect hormone (27 out of 38 DEGs), detoxification enzymes (49 out of 74 DEGs) and digestive enzymes (69 out of 81 DEGs) were downregulated. It is noteworthy that all DEGs associated with chitin synthesis and degradation (8 of 8) and chitin-binding proteins (15 of 15) were downregulated, while all of the 10 DEGs related to endocytosis were upregulated (Figures 4, 5).

### 3.5 Validation of transcriptomic data by qPCR

**FIGURE 6:**
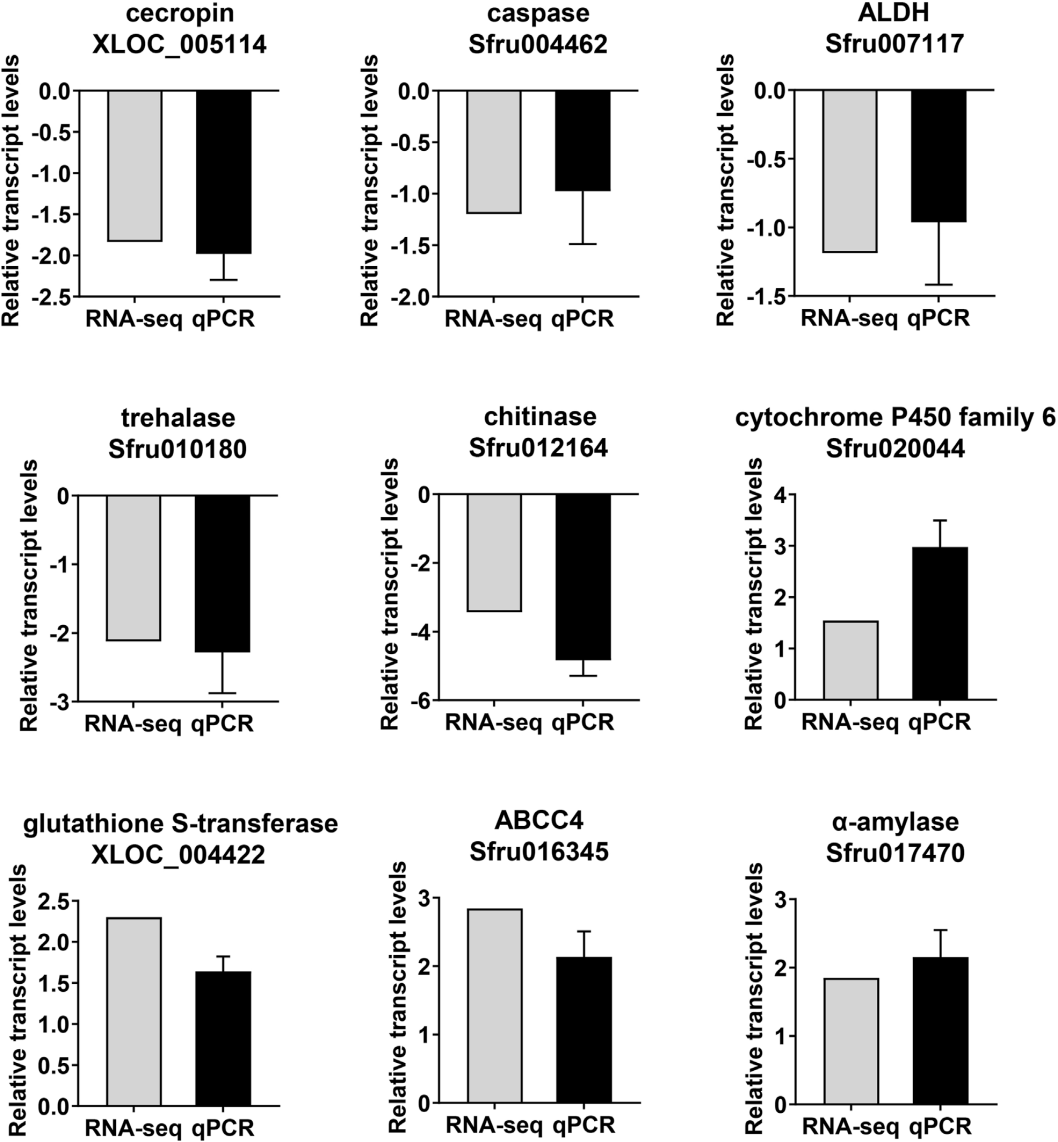
Comparison of expression levels of selected genes in RNA-seq and qPCR.

To validate the RNA-seq results, nine genes were analyzed by qPCR, which are involved in humoral immune response (XLOC_005114 and Sfru004462), insect hormone (Sfru007117) chitin synthesis and degradation (Sfru010180 and Sfru012164) detoxification enzymes (Sfru020044, XLOC_004422 and Sfru016345) digestive enzymes (Sfru017470) were selected and analyzed by qPCR. The qPCR results exhibited a high level of concordance with the transcriptomic data (Figure 6), confirming the reproducibility and reliability of the transcriptomic data.

## 4 Discussion

In this study, the bioassay experiments demonstrated that plumbagin, a natural small molecule naphthoquinone compound in plants, exhibited a high insecticidal activity against the larvae of the distributed destructive lepidopteran pest *S. frugiperda*, consistent with previously reported results that the secondary metabolite has high toxicity to several other lepidopteran pests (Tokunaga et al., 2004; Akhtar et al., 2012a; Rajendra Prasad et al., 2012; Hu et al., 2018; Shang et al., 2019; Dávila-Lara et al., 2021; Rahman-Soad et al., 2021). The findings demonstrated that plumbagin has great potential to serve as a botanical pesticide for controlling second or third instar larvae. However, according to the Globally Harmonized System (GHS) of Classification and Labelling of Chemicals, the toxicity and LC_50_ value of plumbagin against *S. frugiperda* classify it as a Class 4 acute toxicant for humans. This classification may restrict its use as a pesticide. To overcome this limitation, the encapsulation of plumbagin in nanocarriers could be a promising method, enhancing its bioactivity and allowing for reduced dosages and frequencies, thereby effectively minimizing harm to humans and mammals.

To better understand molecular mechanisms underlying the insecticidal effects of plumbagin against *S. frugiperda*, RNA-seq analysis and subsequent qPCR experiments were conducted, which demonstrated that plumbagin treatment could stimulate complex, global transcriptome changes in *S. frugiperda* larvae. Transcriptomic analysis indicated that plumbagin exposure had profound adverse effects on biological and metabolic processes of three nutrients (proteins, lipids and carbohydrates), which are the principal sources of nutrients and energy for insects, and are essential for their normal growth and development (Toprak et al., 2020). Most DEGs related to amino acid transport and metabolism (75 out of 104 genes), lipid transport and metabolism (51 out of 69 genes), and carbohydrate transport and metabolism (43 out of 54 genes) were downregulated (Figures 4, 5). Moreover, most DEGs involved in protein digestion (7 of 8), lipid digestion (13 of 21), and carbohydrate digestion (49 of 52), were downregulated in *S. frugiperda* larvae after plumbagin treatment, which are important for absorption and utilization of these three nutrients. The general suppression of these growth-related genes indicated that plumbagin exposure displayed strong adverse effects on larval growth and development, strongly correlating with bioassay results. Similarly, carvacrol treatment significantly affect the expression levels of genes involved in the metabolism carbohydrates, lipids and proteins in *Lymantria dispar* (Chen et al., 2022).

Transcriptomic analysis showed that plumbagin treatment strongly altered the expression of genes encoding detoxification enzymes/proteins, including *CarEs* (5 up- and 17 downregulated genes), *P450s* (6 up- and 19 downregulated genes), *Glutathione S-transferases* (*GSTs*; 4 up- and 5 downregulated genes), *UDP-glycosyltransferases* (*UGTs*; 5 up- and 6 downregulated genes), *alkaline phosphatases* (2 downregulated genes), and *ABC transporters* (5 upregulated genes). These detoxification enzymes have been reported to play multiple roles in metabolic detoxification of a variety of xenobiotics (Amezian et al., 2021; Giraudo et al., 2015; Li et al., 2007; Vandenhole et al., 2021). For example, the UGT33 family member SfUGT33F28 in *S. frugiperda* is responsible for detoxification of the major maize defensive compound DIMBOA (2,4-dihydroxy-7-methoxy-1,4-benzoxazin-3-one) (Israni et al., 2020). CYP9A subfamily genes in two insect pests *Spodoptera exigua* and *S*. *frugiperda* collectively metabolize two furanocoumarin plant defense compounds (imperatorin and xanthotoxin) and three insecticides (pyrethroids, avermectins, and oxadiazines) (Shi et al., 2023). The carboxylesterase SexiCXE11 in *S. exigua* is able to degrade two plant allelochemicals pentyl acetate and (Z)-3-hexenyl caproate with >50% degradation (He et al., 2020). Although most DEGs responsible for CarEs and P450s were downregulated in *S. frugiperda* larvae (Figures 4, 5), enzyme activity assays revealed that plumbagin treatment led to a significant increase in enzyme activities of both CarEs and P450 (Figure 2). Possible explanations for this phenomenon could be the involvement of post-transcriptional regulatory mechanisms and the contributions of enzyme isoforms with different expression and activity profiles. The inconsistency between RNA-seq data at the transcriptional level and enzyme assay results at the protein level are also reported in previous studies. For example, toosendanin exposure inhibited the expression of most DEGs encoding lipases, while it increased lipase enzyme activity in *S. frugiperda* larvae to overcome the adverse effects of the xenobiotic toosendanin (Lin et al., 2023). Notably, the six upregulated P450s (*Sfru004887*, *Sfru020044*, *Sfru009692*, *Sfru004769*, *Sfru011158* and *Sfru011561*) are mainly concentrated in the CYP6 family and CYP301 family, which are also induced by phytochemicals in other insects, such as *H. armigera* and *Aphis gossypii*, and are associated with plant allelochemical detoxification in insects (Vandenhole et al., 2021). The expression level of one *CYP301A1* gene (*Sfru020044*) were upregulated by 1.55- and 2.97 in RNA-seq and qPCR data, respectively, which was also induced by the phenolic monoterpenoid carvacrol in *S. frugiperda* larvae (Liu et al., 2023). Interestingly, all of five DEGs for the detoxification enzyme ABC transporters (*Sfru016345*, *Sfru007970*, *Sfru007513, Sfru007645* and *Sfru007518*) and all of two DEGs for sodium channel protein (*XLOC_025616* and *Sfru014814*), one type of the insecticide targets, were upregulated in plumbagin-treated larvae, highlighting their potential roles in *S. frugiperda* larvae in response to plumbagin exposure.

Plumbagin treatment led to the downregulation of many DEGs related to humoral immune responses. Almost all DEGs (14 of 15) associated with pattern recognition proteins/receptors including peptidoglycan recognition (*Sfru009140*, *Sfru000940* and *Sfru008126*), protein beta-glucan recognition protein (*Sfru016408*, *Sfru016141*, *Sfru015779* and *Sfru016037*) and c-type lectin including *Sfru000525*, *Sfru015069* and *Sfru004263*, which plays critical roles in initiating humoral immune responses (Tsakas et al., 2010), were downregulated. Besides, several other immune-related genes such as one *scolexin* (*Sfru019519*), one *cecropin* (*XLOC_005114*), four *C-type lysozymes* (*Sfru007716*, *Sfru007030*, *Sfru018720* and *Sfru018779*), two *antimicrobial peptides* (*Sfru000526* and *Sfru000399*), five *peroxiredoxin* (*Sfru009611*, and *Sfru000305*, *Sfru000322*, *Sfru007176* and *Sfru007012*) were also downregulated. Serpin (serine protease inhibitor) is a key negative regulator of melanization responses (Jiravanichpaisal et al., 2006). Almost all DEGs (5 of 6) for serpin, including *Sfru002511*, *Sfru015504*, *Sfru015301*, *Sfru018941* and *Sfru002522*, were downregulated, which suggested that serpin-mediated immunity might be activated by plumbagin treatment. Moreover, all of 10 endocytosis-related genes (*Sfru000635*, *Sfru002360, Sfru009600*, *Sfru013379*, *Sfru014150*, *Sfru015827*, *Sfru022046*, *Sfru002927*, *Sfru002549* and *Sfru019450*), all of 4 *alpha-crystallin* (*Sfru013250*, *Sfru013305*, *Sfru013460* and *Sfru013463*), and almost all DEGs (3 out of 4) involved in JAK-STAT signaling pathway (*Sfru000631*, *Sfru009860* and *Sfru009860*) were upregulated. These findings indicated that plumbagin treatment triggered complex immune responses in *S. frugiperda* larvae.

Cuticle proteins and chitin are the major components of insect cuticle and midgut peritrophic membrane (Shu et al., 2021b). After plumbagin treatment, most DEGs (21 of 27) for insect cuticle proteins, such as *Sfru008849*, *Sfru007124* and *Sfru002859*, and one DEG for skin secretory protein (*Sfru015138*) were downregulated. Similar to our results, Shu et al. (2022) reported that azadirachtin treatment led to the downregulation of many genes for cuticle proteins *S. frugiperda* larvae, whereas the DEGs related to cuticle proteins and skin secretory proteins were upregulated in *P. tsushimanus* larvae after linalool exposure to decrease the penetration of this volatile compound (Li et al., 2023). Besides, plumbagin inhibited the expression of all DEGs (8 genes) involved in chitin synthesis and degradation pathway, including four *trehalase* (Sfru010229, *Sfru010311*, *Sfru009794* and *Sfru010180*), two *chitin deacetylase* (*Sfru018671* and *Sfru018874*) and two *chitinase A* (*Sfru003349* and *Sfru012164*). Trehalase is the first enzyme in chitin biosynthesis, and suppression of trehalase resulted in lethality and morphological defects in several insects (Liu et al., 2019). In present study, all of 4 *trehalases* were downregulated, which might disrupt chitin synthesis and degradation, and consequently led to a decrease in chitin content. Also, all of 15 DEGs associated with chitin-binding proteins (Sfru009934, Sfru010806, Sfru019700, Sfru019167, XLOC_009898, Sfru013067, XLOC_009488, Sfru016397, Sfru019885, XLOC_009577, Sfru010648, Sfru010790, Sfru011009, Sfru018068 and Sfru009342) were downregulated, which play essential roles in forming and maintaining of chitin-containing structures (Tetreau et al., 2015).

## 5 Conclusion

In summary, our results illustrated that plumbagin treatment exhibited a high larvicidal activity against *S. frugiperda* larvae in the bioassay experiments. Consistently, a large number of the DEGs responsible for nutrient and energy metabolism, humoral immune response, insect cuticle protein, chitin-binding proteins, chitin synthesis and degradation, insect hormone, and xenobiotic detoxification were suppressed in RNA-seq data. Conversely, the expression of DEGs involved in endocytosis and the activities of the two detoxification enzymes CarE and P450 were enhanced after plumbagin treatment. These results will help to elucidate insecticidal mechanisms of botanical insecticides against pests.

## Data Availability

The transcriptome sequencing data presented in this study are deposited in the NCBI BioProject database (https://www.ncbi.nlm.nih.gov/bioproject), accession number PRJNA1119741. Further inquiries can be directed to the corresponding author.
