# Supplementary material for: Insecticidal activity and underlying molecular mechanisms of a phytochemical plumbagin against Spodoptera frugiperda
Source: Front Physiol. 2024 Jun 21;15:1427385. doi: 10.3389/fphys.2024.1427385 (PMC11224519; doi:10.3389/fphys.2024.1427385)
Supplement: Supplementary file 1 [file Table1.docx]

**Supplementary material**

**Table S1. Primers used for qRT-PCR analysis in this study.**

| **Name** | **Sequence (5’→3’)** |
| --- | --- |
| XLOC_005114-fwd  XLOC_005114-rev  Sfru004462-fwd  Sfru004462-rev  Sfru007117-fwd  Sfru007117-rev  Sfru010180-fwd  Sfru010180-rev  Sfru012164-fwd  Sfru012164-rev  Sfru020044-fwd  Sfru020044-rev  XLOC_004422-fwd  XLOC_004422-rev  Sfru016345-fwd  Sfru016345-rev  Sfru017470-fwd  Sfru017470-rev  Actin-fwd  Actin-rev | GATGAAGATGATACTGCTGATTAC  CAAGAAGATTGAGAAGATGGG  ACCCACGCTGACTTCCTC  AAGCCACCTTCCTGTTCG  GCTTCGCAAAACAAGTTCG  TGATGCCGTCTTCACCCA  ACCAGCAGTTTGGCGTTAG  CTGTTGTTCGGGCTTTCG  GTCTCGGAGCACCTTCTTA  CTCGTGATACCTTCCAACC  GTCAAGCGTCTGGAAATCG  CTGCCTCCCCAATAACAT  CCTCACCACGGAACAGCA  CGTCAGCGAGCCATAGTC  TGTCCCGTAGTCCAGTGTT  GCACATCCTGCTTGTCATC  TTGGGTGTTGCTGGATTC  GTGGGCAGTGTTGAGGTT  ATCGTTCGTGACATCAAGGAGAAGC  GTTACCGATGGCGATGACCTGAC |

Fwd: forward primer; Rev: reverse primer.

**Table S2. Reads number and genome mapping.**

| **Sample** | **Raw Reads** | **Clean reads** | **Percentage of mapped reads** |
| --- | --- | --- | --- |
| Plumbagin-1 | 43896416 | 42806032 | 80.09% |
| Plumbagin-2 | 45978878 | 45148130 | 80.40% |
| Plumbagin-3  Plumbagin-4 | 41489334  45306896 | 40213076  43803374 | 78.91%  80.91% |
| CK-1  CK-2 | 43908514  45113538 | 42080414  43556366 | 79.24%  79.48% |
| CK-3  CK-4  CK-5 | 44109238  42530904  45306026 | 43223998  40875510  44282348 | 79.10%  77.56%  78.11% |
